# Ethical and Information Governance Considerations for Promoting Digital Social Interventions in Primary Care

**DOI:** 10.2196/44886

**Published:** 2023-09-27

**Authors:** Georgios Dimitrios Karampatakis, Helen E Wood, Chris J Griffiths, Nathan C Lea, Richard E Ashcroft, Bill Day, Neil Walker, Neil S Coulson, Anna De Simoni

**Affiliations:** 1 Wolfson Institute of Population Health Barts and The London School of Medicine and Dentistry, Asthma UK Centre for Applied Research Queen Mary University of London London United Kingdom; 2 Department of Medical Informatics & Statistics The European Institute for Innovation through Health Data Ghent University Hospital Ghent Belgium; 3 The City Law School City University of London London United Kingdom; 4 Medical School Nottingham City Hospital Nottingham United Kingdom

**Keywords:** data governance, digital health, digital intervention, digital social interventions, ethics, information governance, online health communities, peer support, primary care, social intervention

## Abstract

Promoting online peer support beyond the informal sector to statutory health services requires ethical considerations and evidence-based knowledge about its impact on patients, health care professionals, and the wider health care system. Evidence on the effectiveness of digital interventions in primary care is sparse, and definitive guidance is lacking on the ethical concerns arising from the use of social media as a means for health-related interventions and research. Existing literature examining ethical issues with digital interventions in health care mainly focuses on apps, electronic health records, wearables, and telephone or video consultations, without necessarily covering digital social interventions, and does not always account for primary care settings specifically. Here we address the ethical and information governance aspects of undertaking research on the promotion of online peer support to patients by primary care clinicians, related to medical and public health ethics.

## Introduction

The development and progressive adoption of online health social networks, such as on Twitter and Facebook and online health communities (OHCs), has begun to focus attention on their potential to address people’s informational and emotional needs and as services promoted by primary care. Online peer support can enhance effective self-management of long-term conditions, in turn leading to reduced anxiety and improved quality of care, with both direct and indirect health care cost savings [1]. Although digital health interventions do not necessarily result in behavior change [2], evidence from several countries suggests that online peer-to-peer support through OHCs may improve self-management of long-term conditions [3], enhance health-related outcomes [4], and promote adherence to treatment [5]. A review of 11 systematic reviews further emphasizes the potential of online peer support to “improve health outcomes and health-promoting behaviors in targeted populations” [6]. Likewise, a Cochrane systematic review concludes, based on 88 studies (mainly randomized controlled trials), that digital social interventions may result in improvements in health behaviors (eg, steps per day taken or participation in screening tests), in overall health (eg, amount of weight lost or resting heart rate), and in people’s well-being [7].

Welch et al [6] define digital social interventions as “activities among people gathered online who share information using conversational media that make it easy to create and share content in the form of words, pictures, videos, and audios” or as “interventions having an interactive component with 2-way communication between peers or between the website and users.” Strategically promoting online peer support within primary care services as a digital social intervention in primary care has the potential to make a major, sustainable, and global contribution to improving health and reducing the burden of care for patients with long-term conditions on health care providers and health systems [8-10]. Policymakers have started to see the potential of OHCs for improving the self-management of patients at scale. There have been several attempts internationally to promote patient participation in OHCs, for example, the “Togetherall” (previously called the “Big White Wall”), an OHC commissioned by mental health services in the United Kingdom, Canada, and New Zealand [11]; the piloting of closed Facebook groups by the Irish Health System for smoking cessation purposes [12]; and the use of a Facebook group as part of Public Health England’s “Stoptober” smoking cessation campaign [13]. Facebook is also being piloted by National Health Service Digital to promote cancer screening, with promising results [14]. General practice use of open Facebook pages is variable, but most commonly, they have been used to provide generic practice information and to gain patient feedback [15].

Promoting online peer support beyond the informal sector to statutory health services, such as National Health Service primary care, requires ethical considerations and evidence-based knowledge about its impact on patients, health care professionals (HCPs), and the wider health care system. Evidence on the effectiveness of digital interventions in primary care is sparse [16], and definitive guidance is lacking on the ethical concerns arising from the use of social media as a means for health-related interventions and research [17]. Existing literature examining ethical issues with digital interventions in health care mainly focuses on apps, electronic health records, wearables, and telephone or video consultations, without necessarily covering digital social interventions, and does not always account for primary care settings specifically [2,17-24]. Our purpose in this paper is therefore to address the ethical and information governance aspects of undertaking research on the promotion of online peer support to patients by primary care clinicians. Arguments presented here are based on our experience of developing a digital social intervention for patients with troublesome asthma in primary care, the “A Digital Social Intervention for People With Troublesome Asthma Promoted by Primary Care Clinicians” (AD HOC) intervention [25], and relevant material we have found in the literature. We also provide considerations for the formal integration of online peer support into primary care services.

The novelty of this paper lies in considering ethical issues in the context of the primary care setting, impacting a large number of patients who may have long-term or even lifelong relationships with their clinicians. In the AD HOC intervention, primary care clinicians sign patients up to an OHC, thus prompting patients to adopt an OHC-engaged behavior and getting their consent for the OHC engagement to be subsequently analyzed. The primary care setting is unique in that it is the first point of contact for health care purposes across a wider population, through which long-term relationships are formed between clinicians and patients. Therefore, the formal promotion of digital social interventions by clinicians in the primary care setting raises unique ethical issues.

The theory base of the AD HOC intervention draws on the Social Support Theory [26], as modified by Dennis [27] (through Walker and Avant’s [28] concept analysis methodology), applied to health care contexts. Peer support is theoretically embedded within social relationships. Its importance in promoting health and well-being has been recognized. Health promotion through social relationships relies on “shifting responsibility for care to communities” by employing “lay individuals (peers) with experiential knowledge to extend natural (embedded) social networks and complement professional health care services” [29].

Dennis’s [27] conceptual framework relies on the detailed description of relevant concepts encountered in the literature. It defines peer support as “giving of assistance and encouragement by an individual considered equal,” and highlights the diverse interaction modes, settings, providers, and roles through which peer support can be provided, as well as the areas (often referred to as “stressors”) in which peer support is encountered (eg, disease-related, illness-preventing, and health-promoting topics). The conceptual framework goes on by describing the “defining attributes” of peer support interventions, the “effect models” (ie, mechanisms through which peer support interventions lead to health outcomes), “antecedents” (ie, prerequisites for these interventions to work), and by hypothesizing the “potential health outcomes.” The “defining attributes” are summarized as “emotional” (ie, enhancement or restoration of self-esteem), “informational” (ie, provision of “advice, suggestions, factual input, and feedback”), and “appraisal support” (ie, confirmation of the appropriateness of emotions, behaviors, and cognitions). The “effect models,” in turn, consist of the “direct” (ie, direct influence on health outcomes through social integration), “buffering” (ie, buffering impact of stressful events on health), and “mediating effect model” (ie, indirectly impacting health through emotions, cognitions, and behaviors). “Antecedents” refer to the characteristics of the population to receive peer support interventions, selection processes, and training needs for people involved in the delivery of these interventions.

## Ethics

### Overview

For the purposes of this paper, we are using the definition of ethics as described by Denecke et al [21]: “the discipline dealing with what is good and bad and with moral duty and obligation,” and can be classified as medical ethics (focusing on relationships between HCPs and patients) and as public health ethics (focusing on public actions for health and well-being). We highlight here the main ethical matters that emerge from the development and implementation of digital social interventions in primary care. In other words, we are setting out to discern some “good” and “bad” approaches within the “antecedents” of digital social interventions (eg, having primary clinicians introduce and promote online peer support as part of routine health care services), as well as within the “effect models” (eg, when fostering engagement and supporting OHC users, as well as collecting and analyzing data to validate the hypothesized “health outcomes”).

### Matters Relating to Medical Ethics

#### Engagement Importance

Primary care clinicians actively promoting the signing up of patients to established and moderated OHCs and prompting them to interact with other members of that community can be considered a primary care digital social intervention. Patients’ level of engagement with an OHC could be important for gaining benefit, at least for some people, as there is some evidence suggesting that greater OHC engagement translates to improved outcomes [30-35]. Engagement with OHCs, though, is varied, with engagement predictors still being unclear [36]. To maximize engagement with a digital social intervention in primary care, current evidence suggests undertaking developmental work, such as co-designing the intervention with key stakeholders, performing pilot studies, and obtaining feedback on the ideal characteristics of moderators, on use of notifications, and on sharing of discussion topics and resources [17]. Co-designing approaches in health care are important as they set out to “design experiences of patients and staff,” thereby improving “day-to-day experiences of giving and receiving health care” [37]. This is especially true for primary care, a setting with unique features, as pointed out earlier.

#### Looking After Patients Who Engage With OHCs

Two ethical issues emerge from promoting engagement with OHCs in primary care: firstly, the safety of patients and their interactions with existing users of online communities; and secondly, the support by clinicians of those patients who engage themselves with OHCs.

The first point, the safety of OHC interactions, is discussed more in depth later (see “Potential for Harm” section). About the second point, that is, primary care clinicians looking after patients who engage with OHCs, evidence is sparse and concentrated around cyberbullying of patients engaging with social media in general (rather than with OHCs specifically) as well as around identifying misinformation in social media and either correcting it or warning patients accordingly [38-42]. The possibility for social media engagement to negatively affect psychological well-being due to encountering posts with negative feelings (eg, anxiety and worry) is described in the literature [43,44]. Our previous research has indeed highlighted some difficulties faced by highly active OHC users who take on a guiding role (superusers), with their role being stressful at times [45]. Superusers are both patients and caregivers of patients with asthma, have a wide age range, tend to take part in more than one OHC, and spend considerable time in a role sometimes similar to that of moderators. However, most HCPs are unaware of patient-superusers’ engagement with OHCs and are therefore unable to provide support [45]. This is in contrast with the general agreement among superusers that patient engagement with trusted and thriving OHCs should be promoted within health care [45]. Although some people might naturally be drawn to the role of superuser and enjoy the responsibility, this might not always be the case. The lack of support by primary care HCPs might put some superusers off providing advice to peers, causing OHCs to lose the members who significantly contribute to the cohesion of OHCs and the spread of self-management information. Losing OHC superusers and cohesion may mean that primary care patients joining the OHC may not experience the full benefits of online peer support. Our patient and public involvement (PPI) coauthor (BD) has indeed witnessed instances of superusers disappearing from OHCs due to disagreements with other members and high levels of stress.

#### Roles in OHC and Relationships Between Patients and Primary Care Clinicians

The potential for social media to breach boundaries between personal and professional lives and confound roles in health care has been highlighted in the literature [21,46,47]. For example, our previous research revealed a case of 2 retired HCPs, one due to ill health and the other due to age, who assumed the role of OHC superusers [45]. They raised the issue of the need to develop a code of conduct within their registering bodies to engage with users in OHCs. Similarly, signing up to OHCs as users might potentially enable clinicians to “digitally track the personal behavior of patients” [21], including accessing data about patients’ lifestyle, complaints about the care received, or other information not otherwise disclosed to clinicians. However, “spying” on patients is widely recognized as detrimental to the doctor-patient relationship, against the principles of professionalism, and discouraged by existing guidance on social media use by clinicians [48].

With digital interventions in general (not specifically digital social interventions), there are fears that they might “dehumanize” the relationship between HCPs and patients [20,49]. Patients, for example, might increasingly use digital interventions as partial surrogates for information and support that they would have otherwise obtained from HCPs, thereby resulting in fewer interactions and weaker mutual bonds with HCPs. Conversely, participation in an OHC or social media platforms or engagement with digital interventions (in general) might trigger patient queries and requests for subsequent contact with HCPs; hence, promotion of online peer support may end up constituting an extra, time-consuming task for HCPs [20,50,51]. This is important, bearing in mind the increasing workload of clinicians in the UK primary care setting. A survey of OHC members found that patients tend to discuss information they come across in OHCs with their HCPs and that their relationship with HCPs may benefit from OHC engagement due to being more informed about their condition and thus better prepared for consultations [52]. Along the same lines, analysis of an online forum with stroke survivors showed that feedback from online peers facilitated a “distributed deliberation process” (ie, inherent need of patients to discuss with others) and ultimately “shared decision-making with clinicians” (“distributed deliberation” being a prerequisite for “shared decision-making”) [53]. Our PPI coauthor (BD) has indeed confirmed the importance of contacting HCPs before information obtained on the internet from peers is actioned (anecdotal knowledge about different treatment options, for example, when feeling that a condition is being suboptimally managed) and the reassurance that this generates.

### Matters Relating to Public Health Ethics

#### Equality and Diversity in Digital Access

One of the concerns with introducing digital interventions in primary care and in health care in general is their potential to widen health inequalities due to unequal access to digital technologies, associated with differences in patient characteristics [19,20,54-56]. For example, there have been fears that older age groups, ethnic minorities, people with reduced digital and health literacy, those from rural and deprived areas, and those at lower income levels might be excluded from the benefits of digital interventions in primary care (eg, video or email consulting, SMS text messages, mobile health apps, and access to detailed health records). In addition, significantly fewer adults with disabilities engage with the internet compared to people without disabilities [57].

As such, digital social interventions, although removing geographic and physical access barriers as well as some temporal barriers (ie, support is available 24 hours per day rather than restricted to certain times), also have the potential to increase health inequities. However, an overview of systematic reviews of trials looking at the effects of digital social interventions on health outcomes, behavior change, and health equity concluded that OHCs may be effective in promoting health equity [6,7]. Social media interventions have proven not to exacerbate health inequities in certain populations at risk for disadvantage, such as youth, older adults, those with low socioeconomic status, and residents in rural areas [6,7]. Indeed, 70% of homeless people are using social media, and the estimated penetration of broadband connection ownership and the tendency to be influenced by web-based content are wider in ethnic minorities [58]. Analysis of data from a stroke-related OHC found evidence that survivors with a wide range of disabilities successfully engaged with the OHC, and severe disability did not preclude participation [59]. Nevertheless, the concept of “digital inclusion” needs to be at the forefront when adopting digital interventions in primary care, as the risk of the “digital divide” cannot be completely eliminated [60,61], and primary care is usually the major commissioner of health care services and has historically been seen as well positioned to tackle health inequalities [62].

With the AD HOC intervention, our aim is principally to work toward providing evidence that the use of OHCs is indeed beneficial for primary care patients. Proving the benefits of online peer support will encourage primary care clinicians to promote it and patients to engage with OHCs. Although “inclusivity” is not among our primary goals at this early stage, we will seek “digital inclusion” by delivering the AD HOC intervention in areas of London with multiple ethnicities and high levels of social deprivation.

#### Posts From Digital Social Platforms as Data Source for Research

While messages are generally posted publicly (eg, Facebook), some platforms offer the option of posting privately at a one-to-one level (eg, HealthUnlocked [63]). Patients’ public posts in OHCs represent a rich data source to understand the needs, concerns, and preferences of members and to transform posted health information into knowledge that can be disseminated [64]. However, a number of ethical questions pertaining to privacy and informed consent need to be addressed before data from OHCs can be used for subsequent research analyses [65]. Professional bodies such as the British Psychological Society have released ethics-related guidance on internet-mediated research (ie, research involving the remote acquisition of human data through the internet and relevant digital technologies) [66].

#### Privacy and Consent

Paying attention to patient privacy and confidentiality when social media are used in health care is vital due to the possibility of “patient information to be sold” [22]. There have been fears that promoting engagement with OHCs translates to patients being led to “put their views out there” without necessarily being informed (in advance) how their posts will be used [21,22]. The fact that patients seldom read through the terms and conditions of OHCs [22], along with the frequent difficulties patients have in managing their individual privacy settings [67] and regular modifications in OHC privacy policies without notifications [17,22], further restrict comprehension of privacy among OHC members.

OHC privacy is a complicated concept with little consensus on whether OHCs represent private pages or public documents [21,65,66]. In general, members of OHCs do not expect to act as “research subjects” and privacy is among their key concerns [65,68,69]. It has even been noted that although familiarity with a certain social media platform might motivate engagement with OHCs hosted on the same platform (eg, Facebook), there are patients (especially those affected by stigmatized conditions) who prefer to participate in OHCs hosted by platforms that allow maintaining anonymity [17]. However, levels of perceived privacy may vary depending on whether or not an OHC is closed (ie, requiring some sort of registration) and on the membership size, with larger OHCs more likely to be perceived as public spaces [65,70]. OHC moderators and administrators are a good starting point for advice about the attitudes of members toward privacy [66] and, as such, in the AD HOC intervention, we are having regular discussions with staff managing the OHC. Perceived privacy is crucial in determining the need for informed consent before patients are signed up to an OHC and any data produced by their engagement are analyzed. Members of online communities often perceive consent as required when they don’t expect “observation or reporting” of their activity to happen [65].

#### Privacy in Research Studies

Regardless of how privacy is conceived, it is important to ensure the provision of transparent and clear details about data-sharing arrangements to primary care patients invited to participate in OHCs as part of research studies [2]. Within this context of transparency, researchers are advised to monitor the hosting platforms and inform patients who have signed up to OHCs about any substantial changes in terms and conditions or privacy policies [17]. There are increasing suggestions that consent is sought within a “digital/social domain,” especially when data from OHCs are being collected for research purposes (due to possible breaches of anonymity in processing these data) and ideally by offering multiple venues for consenting to ensure patients are aware of what and how their data are being shared [17,66,71]. However, obtaining consent in the sphere of OHCs is not a straightforward procedure, and there have been debates on prospective versus retrospective consenting methods in terms of benefits and drawbacks [65,72,73].

In the AD HOC intervention, we plan to follow a standardized consenting procedure in which primary care clinicians obtain consent at the time of promoting engagement and providing patients with login details to a well-known and moderated OHC after explaining the terms and conditions of the hosting platform to the patient. Face-to-face contact with clinicians will also ensure that competency for consenting is soundly verified, as well as excluding any potential cognitive or other problems that might make them vulnerable to coercion [66]. As part of the consenting procedure in the AD HOC intervention, patients signing up to the OHC are automatically excluded from optional OHC services, such as receiving emails (though they have the option to change these settings themselves by logging into their OHC account).

#### Potential for Harm

As with any digital intervention in primary care, the promotion of online peer support might entail potential risks for both patients’ safety (who could be vulnerable due to their long-term conditions) and the thriving of the wider OHC. These risks are important to be addressed as they might be detrimental to the reputation of primary care clinicians actively promoting OHC engagement. Risks for individuals might arise from (1) untrustworthy health information resources and advice in OHCs—these are common concerns of HCPs in works exploring their attitudes toward OHCs; (2) failure to escalate problems due to a “sense of security” as a result of the OHC-enhanced self-management and due to difficulties in appropriately articulating symptoms when writing posts in OHCs; and (3) social isolation and decreased motivation for engaging in in-person interactions as a result of excessive digital interactions [19,20,45,65]. It should be mentioned, however, that incorrect medical information is usually spotted and removed in established and well-moderated OHCs. Nevertheless, open-ended questions of whether available information is personally relevant and applicable to the individual who is using the OHC still persist. Risks for the viability of OHCs, in turn, might emerge from disruption in the community dynamics triggered by the addition of new patients who do not yet share the norms of “gathering toward a common goal.” Another potential risk could result from the intrusion of clinicians or researchers involved in the promotion of online peer support, who might sign up to the community and “interfere” in OHC communications (eg, to attract participation in research studies), although the latter is only a theoretical risk that has never been encountered [17,21,74]. To mitigate these risks, clinical guidelines for the promotion of digital social interventions in primary care should highlight established and well-moderated OHCs that are safe to be promoted, that is, that have been investigated to ensure their moderation and superusers are sound.

Indeed, our previous research reassuringly revealed that superusers in well-moderated OHCs showed awareness of the complexity of coping with asthma and the limits of their advice. They provided emotional and behavioral self-management support and often directed users back to HCPs for medical queries [45,75]. In addition, researchers’ professionalism and their interest in studying peer-to-peer interactions within an OHC reduce the chances for intrusion and interference in the community’s communications, as any interference might alter the content and type of interactions (eg, formation of networks of communication in the OHC) and thus bias the results of research efforts.

## Information Governance

### Overview

Information governance in health care is related to the strategies used for managing, processing, and sharing data for both direct care and research purposes [76]. Literature highlights concerns about the information governance policies in platforms hosting OHCs, usually run by industry, with inconsistent policies in terms of privacy and security arrangements and “competing commercial and ethical requirements on data ownership and intellectual property,” as well as varying quality standards and ethical oversight [2,77,78].

From our experience of developing a primary care digital social intervention, there are certain information governance matters (relating to data profiling and matching and to the processing of sensitive information or information originating from vulnerable people) that need to be taken into consideration. To begin with, the effects of any systematic (ie, methodical and occurring as per a prearranged strategy) and extensive (ie, involving a large range of data or volume of individuals) profiling of patients need to be considered. Research on the promotion of digital social interventions might involve methodical processes, including data matching (eg, triangulation of self-reported details with data from medical records) by researchers to understand the profile of people likely to benefit from online peer support. Data-matching procedures are also needed to associate activity in the OHC with health-related details, within the context of evaluating the impact of OHC engagement on health outcomes in long-term conditions.

The described data profiling and matching procedures involve the processing of sensitive information (eg, health-related details and activity in the OHC). An additional piece of sensitive information to be collected from patients is their email address, which is necessary to sign them up to the OHC and “tag” their activity for analysis (email addresses are not shared publicly).

However, none of these processes of identifying and signing patients up to the OHC is likely to have a significant impact on individuals, apart from some hesitancy to share personal details. Patients in the AD HOC intervention will decide the amount of data they are willing to share on an individual basis. Only their public interactions in the OHC will be collected and analyzed by the research team.

### Data Confidentiality

Data collected from social networking sites are to be stored behind safe firewalls and ideally outside the original platform [71]. In the AD HOC intervention, patients’ activity in the OHC will be anonymized by its platform host and stored on protected repositories. Any transferring of email addresses and data relating to OHC activity is done on password-protected documents and through encrypted networks. Patients’s email addresses will be stored separately from the data extracted from the OHC.

Postings in OHCs constitute a novel type of data, often referred to as “personal health text” [79]. Risks to confidentiality are inherent in “personal health texts,” as even deidentified formats can be reidentified if combined with other data sets readily available on the internet [66]. Publication of verbatim quotes from “personal health texts” should be avoided as they can be traced to individuals, especially if they contain extensive personal narratives [66]. Bond et al [79] recommend that when “personal health texts” need to be reported in research outputs, data should be aggregated into a single quote summarizing the meaning expressed in different quotes to avoid data being searchable by search engines and to preserve anonymity. Others recommend editing or paraphrasing quotes, but caution is needed not to change the intended meaning [66].

However, there is a lack of clarity on patients’ preferences for anonymity, as some patients may be afraid of being cited, whereas others may seek “publicity,” hence publishing their postings without attribution may be considered a breach of intellectual property rights [21,65,66]. There is also controversy between the need for data openness and the ethical use of “personal health text” [21]. More specifically, while there are arguments that postings in OHCs should be considered as “open data” readily available for download, copying, and analysis without any barriers arising from licensing, others disagree, claiming that “desensitization” of personal information is necessary before any use of OHC data. When “personal health text” is published, by no means should this include “direct patient identifiers (eg, names or pseudonyms, insurance numbers, and photos)” [66,80]. Publication of the name of the OHC researched could also be avoided. However, the disclosure of “up to three indirect patient identifiers” (eg, demographic data such as age, gender, and place of recruitment) is accepted [80].

As the OHC in the AD HOC intervention is considered mostly closed and some members may know the characteristics or views of other members, great care will be taken to pool or aggregate potential identifying information in research outputs. For example, demographic and health-related data will be aggregated and presented as ranges. Only themes and patterns identified in OHC data will be reported. Any reported quotes from postings will be paraphrased to maintain anonymity and will be treated in light of what Eldh et al [81] refer to as an “interpretative approach” (ie, quotations as a means “to bring the text to life—or bring life to the text to highlight particular features of the data while also making the text more appealing to the reader”).

## Implications for Policy and Research

We conclude by summarizing the implications for policy makers, practitioners, and research teams for research on the integration of online peer support into primary care services as a means of enhancing the self-management of patients. There is a need to adhere to “appropriate ethics processes,” ”regulatory processes for digital interventions,” “standards for data handling and sharing,” and guidelines set out by learned societies (eg, the British Psychological Society) at all stages of designing, running, and researching digital social interventions [2]. Baseline work to understand the safety and effectiveness of the target OHCs is required before the promotion of online peer support. The promotion of OHC engagement should be tailored to the preferences and expectations of patients and HCPs. Intuitive user interfaces in the OHCs should be established to encourage participation from patients with a wide range of demographics. The creation of clear instructions for clinicians on how to guide patients appropriately, both when promoting online peer support and in subsequent consultations, will maximize benefits and reduce clinicians’ workload. Guidelines are needed on appropriate phrasing when introducing primary care patients to online peer support, norms and values of engagement with OHCs, on types of need that can be met in OHCs, on cases in which health care support and advice should be sought while engaging with OHCs, and on resources to point patients to. The digital skills of HCPs in primary care [82-84] should be enhanced to increase their understanding of OHCs and their ability to support patients with their participation in OHCs. Patients, in turn, need to be provided with explicit information in relation to privacy arrangements in OHCs. The OHC sign-up of patients should be recorded in medical records to alert the wider primary care team and enable the provision of support, guidance, and timely intervention in case of adverse events.

Tailored discussions with PPI groups about any ethics-related concerns in relation to online peer support and how these concerns can be addressed should take place on an individual study basis.

Information contained in this paper will assist national and international policy makers attempting to strategically foster self-management in primary care patients and inform HCPs and researchers on how to remain on robust ethical grounds when developing, promoting, and studying digital social interventions.

## Abbreviations

AD HOC: A Digital Social Intervention for People With Troublesome Asthma Promoted by Primary Care Clinicians
HCP: health care professional
OHC: online health community
PPI: patient and public involvement
